# Evaluation of Ti/Al alloy coated with biogenic hydroxyapatite as an implant device in dogs’ femur bones

**DOI:** 10.1007/s10856-021-06589-5

**Published:** 2021-09-06

**Authors:** E. M. Mahmoud, M. Sayed, M. Awaad, S. T. El-Zomor, M. Blum, A. Killinger, R. Gadow, S. M. Naga

**Affiliations:** 1 Refractories, Ceramics and building materials Department, National Research Centre, Cairo, Egypt; 2 Department of Surgery, Anaesthesiology and Radiology, Faculty of Veterinary Medicine, Cairo University, Cairo, Egypt; 3 Institute for Manufacturing Technologies of Ceramic Components and Composites (IMTCCC), Stuttgart University, Stuttgart, Germany

## Abstract

The main target of the present research was a full assessment of the toxicity effects and biocompatibility of a Ti/Al-alloy device coated with biogenic hydroxyapatite (bHA) when implanted in dogs in comparison with those of an uncoated Ti/Al-alloy device. The coating of the alloy was carried out using controlled high-velocity suspension flame spray (HVSFS) technique. Both coated and uncoated devices were implanted in dogs’ femur bones for different time periods (45 days and 90 days). Bone-formation ability and healing were followed up, and blood analysis was performed, at Time zero (immediately post surgery), and then at 3 days, 45 days, and 90 days post surgery. Bone mineral density checks, radiological scans of the femur bone, and histological analysis were also conducted. The in-vivo study results proved that implantation of a device made from bHA-coated Ti/Al alloy in dogs’ femur bones is completely safe. This is due to the high osteoconductivity of the coated alloy, which enables the formation of new bone and a full connection between new and original bone material. At 90 days post surgery, the coated alloy had been completely digested within the original bone; thus, it appeared as a part of the femur bone and not as a foreign body. Both the scanning electron microscopy with energy-dispersive X-ray and histology analysis findings affirmed the results. Furthermore, the blood tests indicated no toxicity effects during the 90 days of implantation.

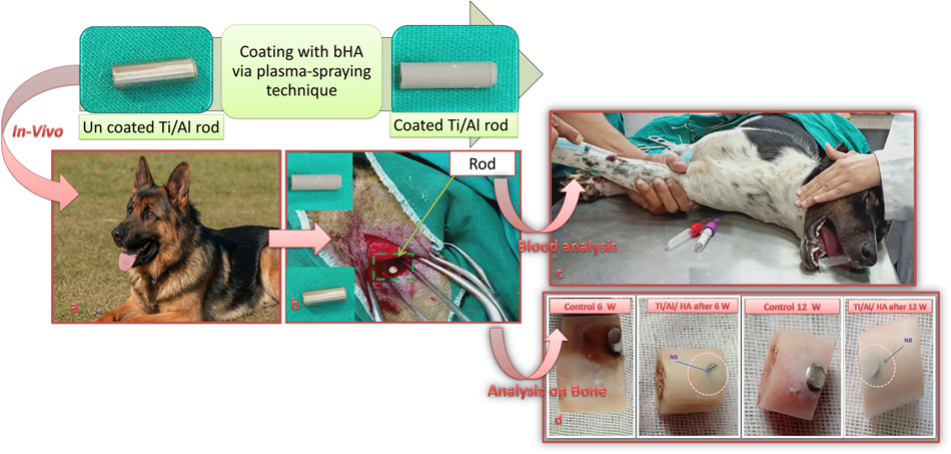

## Introduction

Over the last century, the use of alloys and of alloys coated with bioceramics for the replacement or repairing of bones has led to a range of novel achievements in the field of bone tissue engineering [1]. The fundamental task of an implanted orthopedic device is to fix bone together at a fracture site to fasten new bone formation and reduce bone movement to facilitate the healing process. For optimal outcomes, such implanted devices must be sterilized, biocompatible, bioactive, highly corrosion resistant, nonpoisonous, nonallergenic, and nonmagnetic, and they must have acceptable mechanical properties and a suitable decay mechanism. A variety of materials have been used to make implants, including vanadium (V) and stainless steel, cobalt-based alloys, and titanium-based alloys [2]. Although Ti/Al alloys are inert and widely used for bone substitution, there are some side effects that limit their usage, one of which is the probability of Al and V ions being released into human bodily fluids. These ions can lead to long-term health issues such as allergies, neurogenic damage [3, 4], bone softening, and enzyme function disorders [5, 6]. In addition, the difference in chemical composition between the bone tissue and the implanted alloys results in an inefficient bone-bonding process.

Surface modification of the implanted alloys is essential as bone fixation takes too long with unmodified alloys. Roughing of the surface of the alloys has been adopted as one route to enhance osteoconductivity, to help with new bone formation, and to improve the stability of the implant [2].

Many techniques have been employed for modification of the alloy surface with bioceramics to enhance the mechanical and biological properties of the implants. These techniques include electrochemical deposition [7], hydrothermal electrochemical processing [8], the sol-gel technique [9], high-velocity suspension plasma spraying [10], thermal plasma spraying [11], and electrodeposition [12].

Numerous coating materials have been used to produce modified alloys appropriate for biomedical applications, such as hydroxyapatite, calcium phosphate and calcium silicate, sphene, and bioglass as a bioactive monophasic coating [13–17]. Moreover, some authors investigated the possibility of improving the bonding strength of the coating layer by applying hybrid coating materials such as single-walled carbon nanotubes/hydroxyapatite composite on the Ti alloy via electrochemical deposition technique [18]. Also, the possibility of application of chitosan nanoparticles modified with silver nanoparticle composites on Ti-6Al-4V alloy surface was investigated for enhancing the corrosion protection and the antibacterial activity for the Ti alloy [19]. The application of hydroxyapatite (HA), which is the inorganic component of human teeth and bones, as a coating enhances both the bioactivity and the corrosion resistance of implants made from the alloy Ti6Al4V [20]. It can also increase the osseointegration between the natural bone and the implanted alloy [21, 22].

Many researchers have conducted in-vivo evaluation of alloys coated with HA [23–25]. Suwanprateeb et al. [24] for example, found that screws coated with sol-gel-prepared HA fixed strongly to the natural bones. Compared with uncoated screws, the coated screws displayed superior osteoblast reproduction and mineralization as well as promoting faster bone healing. The premium biocompatibility and fixing strength of HA-coated implants with bone interfaces have also been confirmed in the in-vivo study results published by Golec [23].

The removal of HA-coated Ti screws is completely safe, even after osseointegration. This is a fundamental requirement in case the original surgery fails or corrective procedures are required. In-vivo study results have shown that the HA coating is displaced by newly created bones that implied with the implant surface, while in contrast, a great deal of fibrous tissue is displayed on the surface of uncoated screws. Overall, HA-coated implants display superior mechanical properties to uncoated ones, and furthermore, the HA coating enables the usage of implants for drug delivery [25].

Because high-velocity suspension flame spray (HVSFS) is suitable for coating complex-shaped substrates, we utilized this technique in the present study for coating Ti/Al-alloy devices with biogenic hydroxyapatite (bHA) prepared from fish bones. In our findings, we describe in detail our evaluation of the osteoconductivity and biocompatibility of these coated implants based on in-vivo testing in dogs’ femurs.

## Materials and methods

### Materials

The sample for the present study comprised 20 mongrel male dogs, aged 2–4 years and weighing 18–25 kg. The dogs were randomly distributed into four groups, each containing five dogs. For implantation, 20 cylindrical Ti/Al-alloy rods with a length of 13.0 mm and a diameter of 4.0 mm were used. Ten of the implants were sprayed with a bHA coating, while the other ten remained uncoated.

Of the four groups of dogs, two were control groups: Group I was implanted with uncoated Ti/Al rods for 6 weeks, and Group II was implanted with uncoated Ti/Al rods for 12 weeks. The other two groups were the treated groups: Group III was implanted with bHA-coated Ti/Al rods for 6 weeks, and Group IV was implanted with bHA-coated Ti/Al rods for 12 weeks.

### Methods

#### The sample preparation and sterilization

10-rod Ti-6Al-7Nb (TAN) samples that had a diameter of 4 mm and lengths of 13 mm, which approved by the US FDA and supplied by (DePuy Synthes, USA), were used as control implant samples. Other 10-rod samples of Ti-6Al-7Nb (TAN) coated with bHA were used for implantation as implanted coated samples. Natural Hydroxyapatite powder was extracted from fishbone skeletons by heat treatment procedure according to the method described by Naga et al. [26]. The obtained hydroxyapatite powder was used as a bio-material coat on the Ti-6Al-7Nb (TAN) rods via a controlled HVSFS technique. The obtained bHA-coat over the Ti-6Al-7Nb alloys had an average coating thickness of ≈about (22.8 µm), whereas the average surface roughness was almost 1.40 µm.

The sterilization process was carried out by an autoclave, which uses steam under pressure to kill harmful bacteria, viruses, fungi, and spores that may be contaminated the samples. The uncoated Ti/Al rods and bHA-coated Ti/Al rods were sterilized for a given amount of time (20 min) and the temperature for steam sterilization was 275 °F (135 °C). The sterilization process didn’t affect the hydroxyapatite (HA) layer due to the high thermal stability of the hydroxyapatite coat, which reaches 1200 °C compared to the low sterilization temperature (135 °C).

#### Surgical intervention

The present study was carried out in conformity with the Ethical Guidelines for Animal Care and the guidance of the Ethics Committee of the National Research Center of Egypt with approval number (ACEC#: 15/039).

**Fig. 1:**
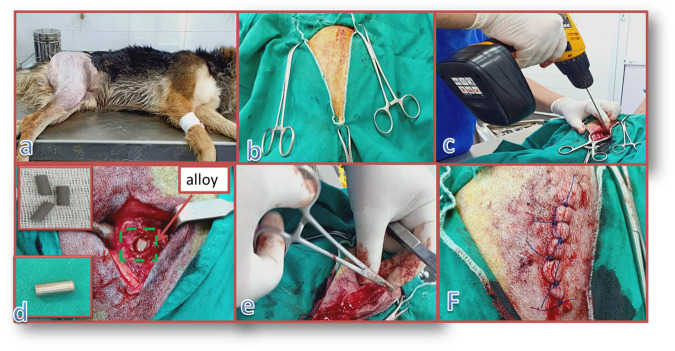
Surgical procedures: **a** shaving the dog and sterilizing the alloy implant, **b** opening the skin and muscles, **c** drilling into the femur bone of the dog, **d** implantation of the alloy rods in the femur bone opening, **e** starting to suture the muscles, and **f** completed suturing of the two sides

Each dog was prepared for surgery by fasting and by shaving of the operation area, which included the area of the femur from the mid-dorsal line to the mid-tibia. Anesthesia was applied using atropine and xylazine as premedications and ketamine HCl or sodium thiopental for induction and maintenance. A surgical incision of ~10 cm was made on the lateral aspect of the thigh, passing through the subcutaneous tissue and tensor fascia lata, and then the vustus lateralis and biceps femoris muscles were bluntly separated to expose the femur. A hole was then drilled in the proximal femur. The drilled holes have diameters of 4 mm and lengths of 13 mm. The implants have exactly the same dimensions as the drilled holes. After drilling, the defect was extensively washed with saline to evict bone remains. The implant was then hammered into the hole. The surgical wound was closed routinely, and each dog was injected with diclofenac sodium and cefotaxime sodium for 5 days to control postoperative pain [27]. At 6 or 12 weeks after implantation (according to group), the dogs were sacrificed to allow for evaluation of the results. Clinical findings, in-vivo osseointegration evaluation, biochemical analysis and histological analysis, bone mineral density (BMD) evaluation, radiograph analysis, and statistical analysis were performed for all animals in the lateral and anteroposterior (AP) positions to identify any bone changes during the period of the experiment (0, 6, and 12 weeks). Figure 1 shows the surgical procedures.

### Characterization

BMD g/cm^3^ and bone mineral content (BMC) were measured using the DEXA Sabre and Sabre Research software (Norland Medical Systems Inc., XR-46, Fort Atkinson, Wisconsin, Model 434 A063, USA). To carry out BMD measurement, all the dogs were sacrificed at the end of the experiment (6 or 12 weeks post surgery dependent on group). The femur bones for each group were analyzed and as an internal standard to avoid inter-scan variations the femur measurements were performed on a 1-cm-thick Plexiglas table. All bones were measured in the same scanner using a high-resolution setting with line spacing set at 0.01 cm. Radiological analysis was carried out using a mobile X-ray apparatus (HP machine) at the Department of Surgery in the Faculty of Veterinary Medicine at Cairo University. A histological sample was extracted from each group from within the region of bone regeneration in the femur. The samples were fixed with 10% neutral formalin, embedded in paraffin after decalcification, and stained with hematoxylin and eosin as a routine stain for cellular details. All stained specimens were checked by naked eye and under a light microscope (Olympus BX61, Hamburg, Germany) connected to a high-resolution digital camera (Olympus, E330, Imaging Corp). The microstructure of the samples was investigated using a scanning electron microscope (SEM) (Model XL30, Philips, Eindhoven, Netherlands) coupled with Energy-Dispersive spectroscopy (EDXs).

### Statistical analysis

Statistics were calculated using the software program IBM SPSS Statistics Version 21.0, (United States USA) and the data presented as Mean ± Standard Deviation. Comparison between two groups was performed using independent *t*-test and *p* value was considered statistically significant if ≤0.05.

## Results

### Clinical findings

**Fig. 2:**
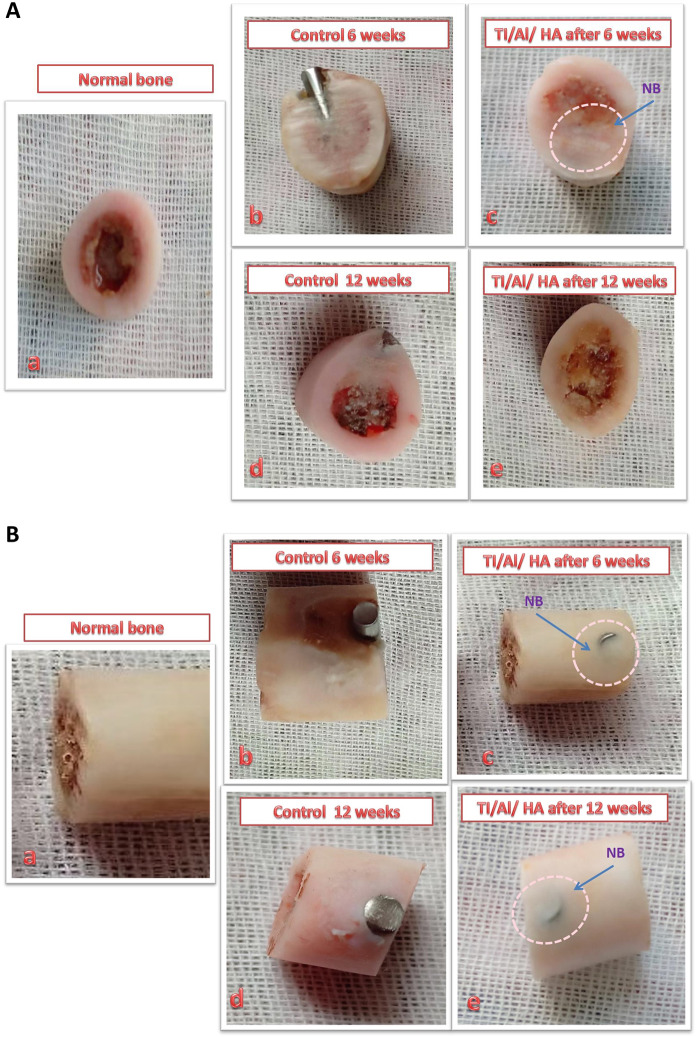
**A** Cross-sections of femur bones: (a) normal, (b) control group after 6 weeks (Group I), (c) treated group after 6 weeks (Group III), (d) control group after 12 weeks (Group II), and (e) treated group after 12 weeks (Group IV). **B** Femur bones: (a) normal, (b) control group after 6 weeks (Group I), (c) treated group after 6 weeks (indicating newly formed bone [NB]) (Group III), (d) control group after 12 weeks (Group II), and (e) treated group after 12 weeks (proving the formation of new bone [NB]) (Group IV)

It has to be stated that the difference between the control group (G1) and group (GII) is a time difference. GI: refers to the femur bone of doges implanted with uncoated Ti/Al rods for 6 weeks, while GII refers to the femur bone implanted with the uncoated Ti/Al rods for 12 weeks. On comparing between the two groups it was found that in group I: the drilled hole was still opened and the inner part of the hole defect is still present after 6 weeks post surgery. For Group II nearly most of the drilled hole was covered with newly formed bone and the open hole defect was still opened (12 weeks post operations). Figure. 2

The findings presented in Fig. 2 show that the bone defect in Group III (coated implants, 6 weeks) was nearly healed. Moreover, they indicate that the newly formed bone was almost filling the inner part of the defect by the 6-week time point. A remarkable amount of new bone fragments were also present in the outer part of the femur bone of the dogs at this point, as seen in Fig. 2A. At 12 weeks post surgery (Group IV), the bone defect was completely healed and filled with newly formed bone. The healed femur had become a normal bone, as shown in Fig. 2B.

### Biochemical analysis

Post surgery, all the studied dog groups were tracked using biochemical markers for safety evaluation of the coated and uncoated implants. The studied biochemical indicators were as follows: (1) kidney function, (2) liver function, (3) tumor markers, (4) denoting of free radicals, (5) inflammatory effects, and (6) hematology.

#### Kidney function

##### Serum CRE levels

Table 1 demonstrates the serum CRE levels for the control groups (I and II) and the treated groups (III and IV). The statistical analysis indicated that the serum CRE levels in the Group III and Group IV dogs were lower than those in the corresponding control groups. However, the differences were not statistically significant (*p* > 0.05), with *p*-values of 0.09 and 0.41, respectively. The normal CRE level for dogs is [0.5–1.6 mg/dl] [28]. These findings indicate that the implants did not affect the dogs’ kidney function.

**Table 1:** Serum CRE and serum urea levels (mg/dl), ALT, AST, and ALP levels (U/L), arginase (U/L), AFU (U/L), and GSH, SOD (U/mL), LIP (nmol/mL), and NO (nmol/L) of the treated group dogs (bHA-coated) (Groups III and IV) compared with the control group (uncoated) dogs (Groups I and II) at T0 (immediately post surgery) and at 3, 45, and 90 days post surgery

Test	Time, days	Implantation time, weeks					
		45			90		
		Control (uncoated) (Group I)	Group III (bHA-coated)	Sig. value	Control (uncoated) (Group II)	Group IV (bHA-coated)	Sig. value
CRE (mg/dl),	**0**	0.71 ± 0.15	0.70 ± 0.11	0.72	0.82 ± 0.03	0.62 ± 0.04	0.51
	**3**	0.71 ± 0.06	0.60 ± 0.15	0.22	0.76 ± 0.16	0.59 ± 0.03	0.37
	**45**	0.69 ± 0. 59	0.60 ± 0.13	0.09	0.66 ± 0.02	0.56 ± 0.05	0.25
	**90**				0.63 ± 0.01	0.50 ± 0.07	0.41
UREA (mg/dl)	**0**	25.40 ± 0.51	22.40 ± 0.75	0.415	25.6 ± 0.49	25.40 ± 0.43	0.603
	**3**	24.0 ± 0.36	21.80 ± 0.61	0.085	28.40 ± 3.52	25.60 ± 1.47	0.071
	**45**	21.60 ± 0.69	20.80 ± 0.63	0.833	29 ± 0.54	22.40 ± 0.45	0.747
	**90**				29 ± 0.71	20.41 ± 1.63	0.13
ALT (u/l)	**0**	40.40 ± 4.057	43.80 ± 1.65	0.056	49 ± 0.34	40 ± 0.27	0.561
	**3**	30.40 ± 2.87	44.86 ± 2.17	0.938	35.60 ± 3.93	33.20 ± 0.54	0.298
	**45**	37 ± 0.641	40 ± 0.58	0.280	40 ± 2.63	38.60 ± 1.98	0.124
	**90**				34.20 ± 1.24	37.40 ± 1.69	0.69
AST (u/l)	**0**	24.64 ± 14.52	26.86 ± 1.86	0.703	21.2 ± 0.18	17.14 ± 0.86	0.087
	**3**	19.68 ± 1.45	24.5 ± 1.14	0.477	20.4 ± 0.76	18.6 ± 0.25	0.083
	**45**	24.96 ± 1.28	25.1 ± 1.59	0.524	28.94 ± 3.18	25.2 ± 1.32	0.081
	**90**				44 ± 1.58	40 ± 1.61	0.13
ALP (u/l)	**0**	30.68 ± 2.28	28.0 ± 34.90	0.227	29.82 ± 1.76	30.48 ± 1.93	0.645
	**3**	28.72 ± 2.38	25.66 ± 6.23	0.382	26.3 ± 0.88	26.78 ± 0.11	0.801
	**45**	37.08 ± 3.6	36.62 ± 1.90	0.93	31.22 ± 0.29	42.60 ± 0.98	0.094
	**90**				60.00 ± 0.16	91.00 ± 0.2	0.074
Arginase(u/l)	**0**	8.20 ± 0.09	8.06 ± 0.26	0.216	6.94 ± 0.32	6.54 ± 0.40	0.336
	**3**	8.26 ± 0.46	5.98 ± 0.98	0.134	9.080 ± 0.24	7.08 ± 0.39	0.95
	**45**	9.24 ± 0.36	7.28 ± 0.89	0.157	9.90 ± 0.32	7.00 ± 0.57	0.422
	**90**				7.80 ± 0.58	6.92 ± 0.31	0.132
AFU (u/l)	**0**	0.89 ± 0.08	0.79 ± 0.79	0.089	0.57 ± 0.05	0.41 ± 0.03	0.167
	**3**	0.64 ± 0.05	0.54 ± 0.09	0.123	0.85 ± 0.04	0.58 ± 0.06	0.95
	**45**	0.79 ± 0.03	0.62 ± 0.04	0.367	0.92 ± 0.02	0.80 ± 0.03	0.387
	**90**				0.91 ± 0.03	0.77 ± 0.01	0.156
GSH (nmol/ml)	**0**	13.70 ± 0.55	14 ± 1.07	0.45	12.64 ± 0.67	12. 98 ± 0.68	0.73
	**3**	14.56 ± 0.50	15.14 ± 1.10	0.24	16.16 ± 0.22	17.70 ± 0.83	0.74
	**45**	15.14 ± 0.56	16.02 ± 0.11	0.34	17.16 ± 0.35	18.7 ± 0.40	0.63
	**90**				14.84 ± 0.67	19.00 ± 0.71	0.82
SOD (U/mL)	**0**	4.26 ± 0.39	4.20 ± 0.39	0.85	5.06 ± 0.32	4.34 ± 0.30	0.77
	**3**	4.78 ± 0.66	5.52 ± 0.64	0.90	5.74 ± 0.08	4.16 ± 0.37	0.06
	**45**	6.42 ± 0.16	5.46 ± 0.69	0.23	7.22 ± 0.13	5.74 ± 0.69	0.07
	**90**				5.64 ± 0.52	5.40 ± 0.27	0.17
LIP(nmol/ml)	**0**	4.14 ± 0.29	4.02 ± 0.86	0.08	4.7 ± 1.65	3.42 ± 0.42	0.49
	**3**	7.9 ± 0.22	7.44 ± 0.37	0.28	4.78 ± 0.17	3.60 ± 0.68	0.47
	**45**	7.32 ± 0.37	6.44 ± 0.09	0.19	8.38 ± 0.23	7.38 ± 0.73	0.91
	**90**				7.00 ± 0.51	6.44 ± 0.62	0.43
NO (nmol/l)	**0**	15.40 ± 0.46	15.24 ± 0.56	0.56	17 ± 0.38	15.26 ± 0.38	0.87
	**3**	18.40 ± 1.07	15.80 ± 1.88	0.13	20.00 ± 0.70	16.56 ± 1.68	0.31
	**45**	20.00 ± 0.25	18.40 ± 0.16	0.32	20.00 ± 1.58	14.20 ± 1.39	0.59
	**90**				18.94 ± 0.61	15.00 ± 0.08	0.19

##### Serum urea analysis

The serum urea levels for all the groups of dogs are shown in Table 1. We note that the urea levels were lower in the serum of Group III and Group IV than in the corresponding control groups; however, the differences were not statistically significant (*p* > 0.05). The normal urea level of dogs is [8.7–30.5 mg/dl] [29].

#### Liver functions

The liver function indicators measured in this study were alanine aminotransferase (ALT), aspartate aminotransferase (AST), and alkaline phosphatase (ALP) levels in the blood serum.

##### Detection of ALT in the blood serum

The results showed that implantation of bHA-coated Ti/Al alloy in the femur bones of dogs for 6 weeks (Group III) showed increased ALT activity compared with the control group (Group I); however, the difference was not statistically significant (*p* > 0.05). In contrast, after 12 weeks of implantation (Group IV), ALT activity was decreased in comparison with the corresponding control group (Group II). This difference was also not statistically significant (*p* > 0.05) at *p* = 0.69 (Table 1). The normal range of ALT for dogs is [5–107 U/L] [28].

##### Detection of AST in the blood serum

The results indicated that, at 6 weeks, the AST level in the dogs implanted with the coated alloy (Group III) was higher than in the control group (Group I). The difference was not significant (*p* > 0.05) at *p* = 0.524. On the other hand, Group IV (12 weeks) showed a decrease in AST activity compared with the corresponding control group. Again, the difference was non-significant (*p* > 0.05) at *p* = 0.13 (Table 1). The normal AST level for dogs is [5–55 U/L] [30].

##### Detection of ALP in the blood serum

The ALP activity in the serum of the dogs treated with the coated alloy for 6 weeks (Group III) was lower than that in the corresponding control group (Group I). Statistical analysis indicated that the difference was not significant (*p* > 0.05) at *p* = 0.93. On the other hand, after 12 weeks of implantation (Group IV), the coated implants led to higher ALP activity than in the control group (Group II) at a level of *p* = 0.074, which was not statistically significant (*p* > 0.05).

#### Tumor markers

##### Arginase activity

Table (1) illustrates the arginase levels (U/L) for dogs in the treated groups compared with the control groups. The table shows that the arginase levels in Groups III and IV were lower than in the control groups. The differences were not statistically significant (*p* > 0.05) at levels of *p* = 0.16 and 0.13, respectively.

##### α-L-fructosidase activity

The results in Table (1) show that implantation of the coated alloy in the femur bones of dogs for either 6 or 12 weeks resulted in lower α-L-fructosidase (AFU) activity than in the corresponding control groups. Statistically, the differences were not significant (*p* > 0.05) at *p* = 0.367 and 0.156, respectively.

##### Reduced glutathione measurement

The effect of implantation of the coated alloy on the reduced glutathione (GSH) activity in the blood serum of the dogs is shown in Table 1. There was higher GSH activity in Groups III and IV compared with the corresponding control groups, at *p*-values of 0.34 and 0.82, respectively. These differences were thus not statistically significant (*p* > 0.05).

#### Free radical biomarkers

##### Super-oxide dismutase

Table 1 shows the super-oxide dismutase (SOD) levels (U/mL) of the treatment group dogs in comparison with the control group dogs. At 6 weeks, the SOD levels in the treated dogs’ blood serum showed a slight change compared with the control groups, while after 12 weeks, the level was decreased. The abovementioned changes were not significant, at *p* = 0.23 and 0.17, respectively.

##### Lipid peroxides (malondialdehyde)

The results shown in Table 1 indicate that implantation of the coated alloy in the femur bones of the dogs for 6 and 12 weeks resulted in decreased lipid peroxide (LIP) activity compared with the corresponding control groups, but the differences were not statistically significant (*p* > 0.05) at *p* = 0.19 and 0.43, respectively.

#### Inflammatory effects

The results of the implantation of the coated alloy showed that nitric oxide activity was decreased at both 6 and 12 weeks. In both cases, the differences between nitric oxide (NO) activity for the animals in the treated groups and the corresponding control groups were not statistically significant (*p* > 0.05) (Table 1).

#### Hematology

The toxicity effects in the control groups and the treated groups were evaluated by measuring white blood cells (WBCs), red blood cells (RBCs), platelet count (PLT), and hemoglobin levels at T0 (immediately post surgery) and at 3-, 45-, and 90-days post surgery. As shown in Table (2), the results indicated higher hemoglobin, RBC, and PLT levels in the treated groups than in the control groups. The complete blood count (CBC) findings were normal, while a decrease in WBCs was observed. Table 3 shows an increase in the WBC, RBC, and PLT counts and the HB levels of the implanted dogs at 3 days post surgery. There was no significant effect on the measured parameters, and the CBC findings were normal. Table 4 shows that, after 45 days of the implantation, the RBC, WBC, and PLT counts of the treatment group dogs were higher than those of the dogs in the control groups. Meanwhile, the hemoglobin counts were lower in the treated groups than in the control groups. In general, no significant toxicity effects were observed, and normal blood results were recorded.

**Table 2:** Toxicity effects on RBCs, WBCs, hemoglobin, and PLT count at T0

Samples symbol	WBCs (*n* × 10^3^) normal: (5.50–19.5)	RBCs (*n* × 10^6^) normal: (5.7–10.5)	HB g/dl normal: (9–16)	PLT (*n* × 10^3^) normal: (160–420)
Control (un coated) (Group I)	18.02 ± 0.71	6.85 ± 0.33	14.80 ± 0.58	165.00 ± 0.45
Group III (bHA-coated)	15.48 ± 1.18 *P* = 0.09	7.52 ± 0.50 *P* = 0.08	15.10 ± 0.12 *P* = 0.06	304.00 ± 0.50 *P* = 0.13
Control (un coated) (Group II)	17.80 ± 1.39	6.62 ± 0.39	14.04 ± 0.16	170.00 ± 0.71
Group IV (bHA-coated)	14.60 ± 0.95 *P* = 0.12	7.08 ± 0.50 *P* = 0.22	15.86 ± 0.43 *P* = 0.87	326.20 ± 0.36 *P* = 0.93

**Table 3:** Toxicity effects on RBCs, WBCs, hemoglobin, and PLT count at 3 days post surgery

Samples symbol	WBCs (*n* × 10^3^) normal: (5.50–19.5)	RBCs (*n* × 10^6^) normal: (5.7–10.5)	HB g/dl normal:(9–16)	PLT (*n* × 10^3^) normal:(160–420)
Control (un coated) (Group I)	17.16 ± 0.74	6.70 ± 0.51	14.20 ± 0.75	182.00 ± 0.61
Group III (bHA-coated)	18.64 ± 1.86 *P* = 0.85	6.95 ± 0.25 *P* = 0.23	15.18 ± 0.84 *P* = 0.59	232.0.63 *P* = 0.62
Control (un coated) (Group II)	11.50 ± 0.58	6.80 ± 0.85	14.42 ± 0.59	312.00 ± 4.72
Group IV (bHA-coated)	15.02 ± 1.08 *P* = 0.92	7.5 ± 0.51 *P* = 0.095	14.60 ± 0.72 *P* = 0.268	258 ± 0.41 *P* = 0.213

**Table 4:** Toxicity effects on RBCs, WBCs, hemoglobin, and PLT count at 45 days post surgery

Samples symbol	WBCs (*n* × 10^3^) normal: (5.50–19.5)	RBCs (*n* × 10^6^) normal: (5.7–10.5)	HB g/dl normal:(9–16)	PLT (*n* × 10^3^) normal: (160–420)
Control (un coated) (Group I)	12.20 ± 0.94	7.80 ± 0.55	15.76 ± 0.57	225.8 ± 0.19
Group III (bHA-coated)	12.40 ± 0.74 *P* = 0.876	8.90 ± 0.91 *P* = 0.96	14.06 ± 0.773 *P* = 0.09	272.00 ± 0.27 *P* = 0.16
Control (un coated) (Group II)	13.30 ± 0.72	8.50 ± 0.49	15.30 ± 0.54	180.00 ± 0.34
Group IV (bHA-coated)	19.20 ± 1.93 *P* = 0.98	9.64 ± 0.39 *P* = 0.18	14.8 ± 0.55 *P* = 0.09	320.00 ± 0.21 *P* = 0.06

The results for 90 days post surgery are shown in Table 5. The HB levels and RBC counts were diminished, while increases were observed in both the WBC and PLT counts. The changes were within the normal ranges for dog blood.

**Table 5:** Toxicity effects on RBCs, WBCs, hemoglobin, and PLT count at 90 days post surgery

Samples symbol	WBCs (*n* × 103) normal:(5.50–19.5)	RBCs (*n* × 106) normal: (5.7–10.5)	HB g/dl normal :(9–16)	PLT (*n* × 103) normal: (160–420)
Control (un coated) (Group II)	10.70 ± 0.12	6.50 ± 0.20	14.34 ± 0.39	178.00 ± 0.33
Group IV (bHA-coated)	13.08 ± 1.43 *P* = 0.18	5.06 ± 0.31 *P* = 0.23	12.09 ± 0.50 *P* = 0.48	180.00 ± 1.04 *P* = 0.56

##### Bone mineral density

BMD is a widely used variable for diagnosis and observation of skeletal defects such as osteoporosis and osteopetrosis [31–33]. Applying BMD analysis at central sites in the skeleton permits the recording of disease progress and the monitoring of alterations that occur in the bones during the healing process.

The BMD findings for bone defects implanted with the uncoated and the coated alloy, whether for 6 weeks or 12 weeks, appeared normal at all time points. At 6 weeks post surgery, the bone was not completely restored at the defect site; however, the treatment groups (coated with bHA) showed higher BMD than the control groups.

**Fig. 3:**
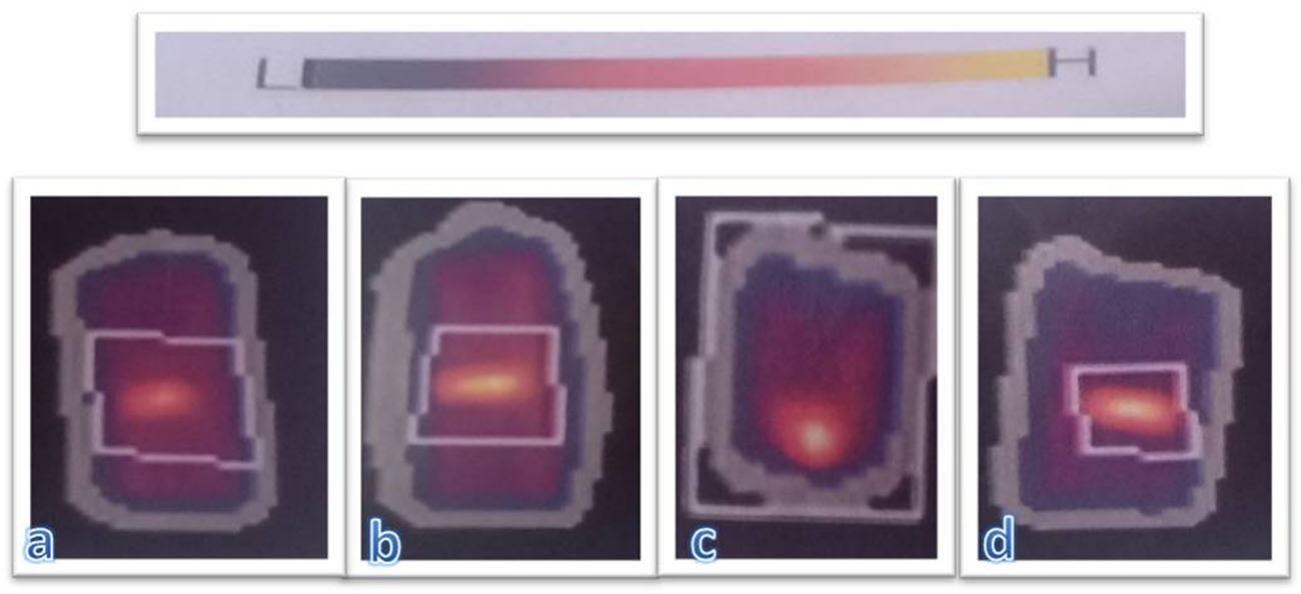
This figure refers to DEXA scan image of the femur bone of the dogs (**a**) control group I (**b**) group III (**c**) control group II (**d**) group IIII. The relative areal of bone mineral density (BMD) is indicated by: H high density, L low density appearance of yellow color indicate high bone mineral density of femur bone, range on the bar change from dark color to yellow color taken from instrument

Figure 3 refers to the DEXA scan image of the femur bone of the dogs. (a) is for the control group I, (b) for group III, (c) for control group II, and (d) for group IIII. The relative areal of BMD is indicated by: H = high density, L = low density. The appearance of yellow color indicates high BMD, while the dark color refers to low BMD of the bone.

At 6 weeks post surgery, the statistical analysis showed that the recorded differences between the BMD and BMC of the grafted bone defects and the control bone samples were not significant (*p* > 0.05) at *p* = 0.35 and 0.29, respectively. After 12 weeks, the differences had increased, but they were still not significant (*p* > 0.05) at *p* = 0.10 and 0.279, respectively.

### Radioactive analysis

**Fig. 4:**
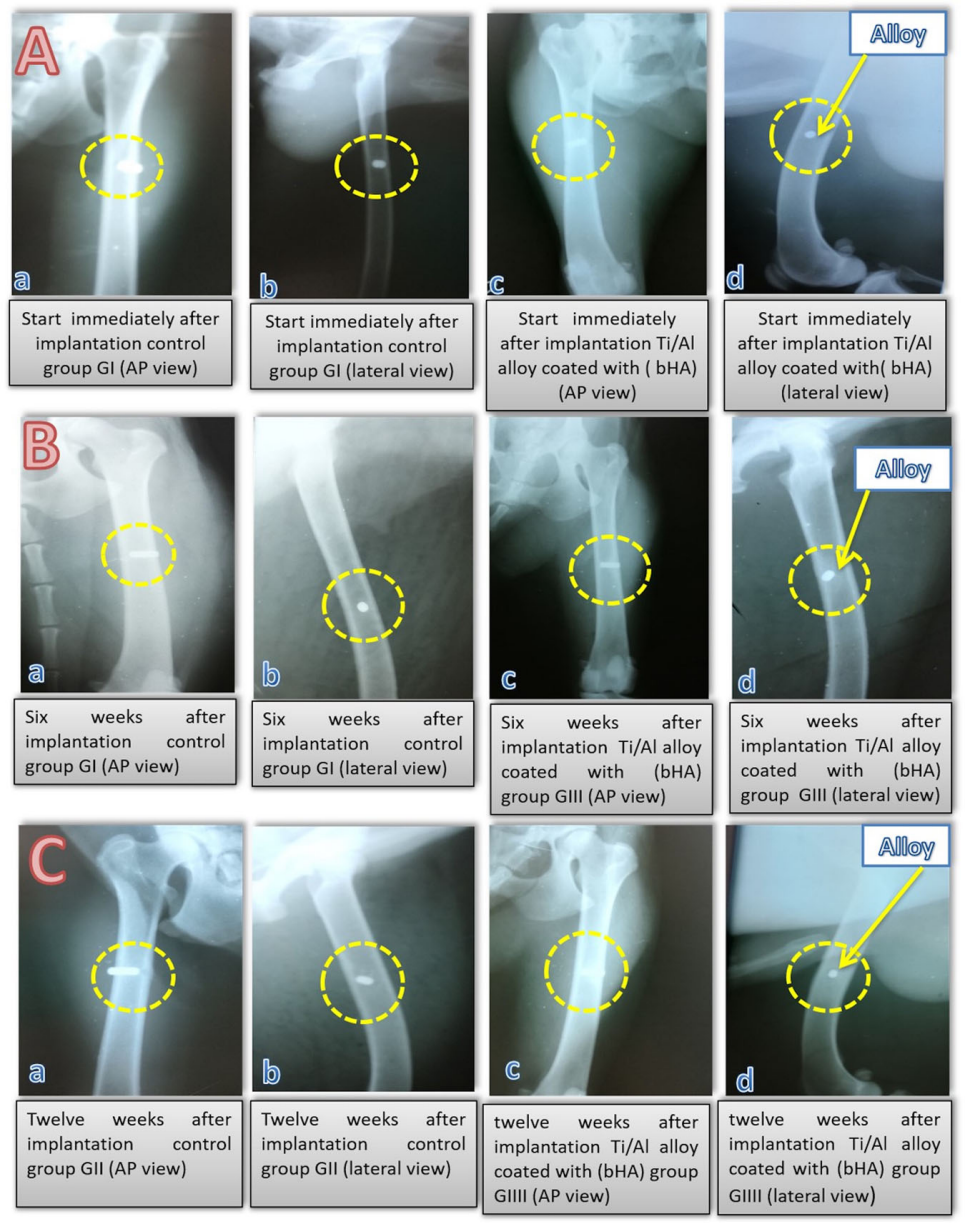
This figure is the radiographic images for: **A** Control Group (un- coated) and b-HA coated Group (treated) for all four groups (in both the AP and lateral view) immediately after implantation, at zero time (T0). **B** Control Group I and treated Group III (in both AP and lateral view) after 6 weeks. **C** Control Group II and treated Group IV (in both AP and lateral view) after 12 weeks

The radiographic images for Group III after 6 weeks revealed increased opacity surrounding the coated implant in comparison to the images for the control group (Group I) as in Fig. 4B and from Time zero (T0) Fig. 4A). The radiographic images for Group IV after 12 weeks revealed that there was an area of opacity filling the entire medullary cavity surrounding the coated implants; this area was larger than that seen in the images for the control group (Group II) as in Fig. 4C.

### Scanning electron microscope and energy-dispersive X-ray spectroscopy

#### For 6 weeks post surgery

##### Group I results

**Fig. 5:**
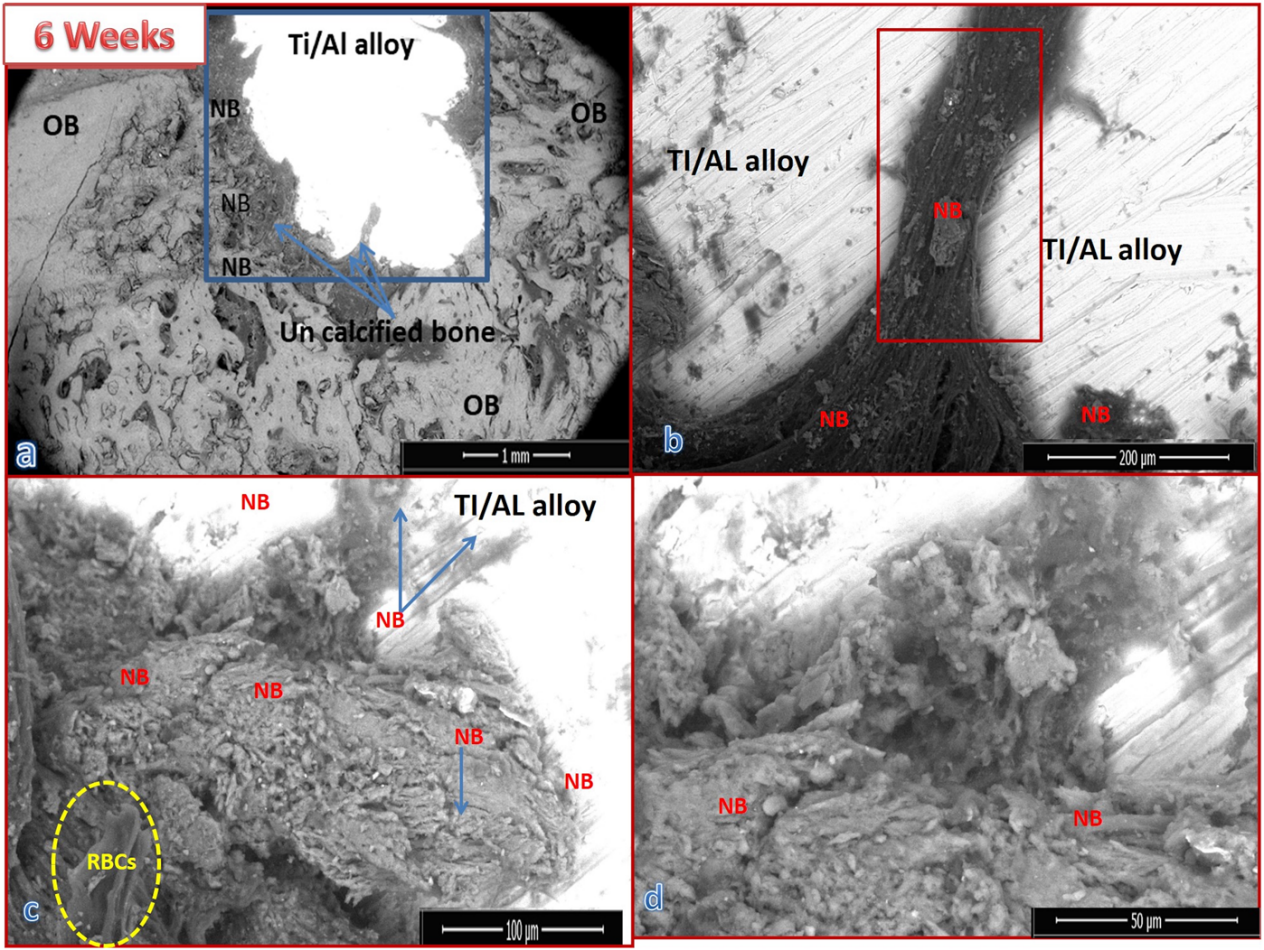
SEM micrographs with different magnifications for a cross-section of bone defect drilled in the dog femurs and grafted with uncoated Ti/Al alloy (control group) for 6 weeks: **a** at ×60 magnification, b at ×600 magnification, **c** at ×1200 magnification, and **d** at ×2400 magnification

**Fig. 6:**
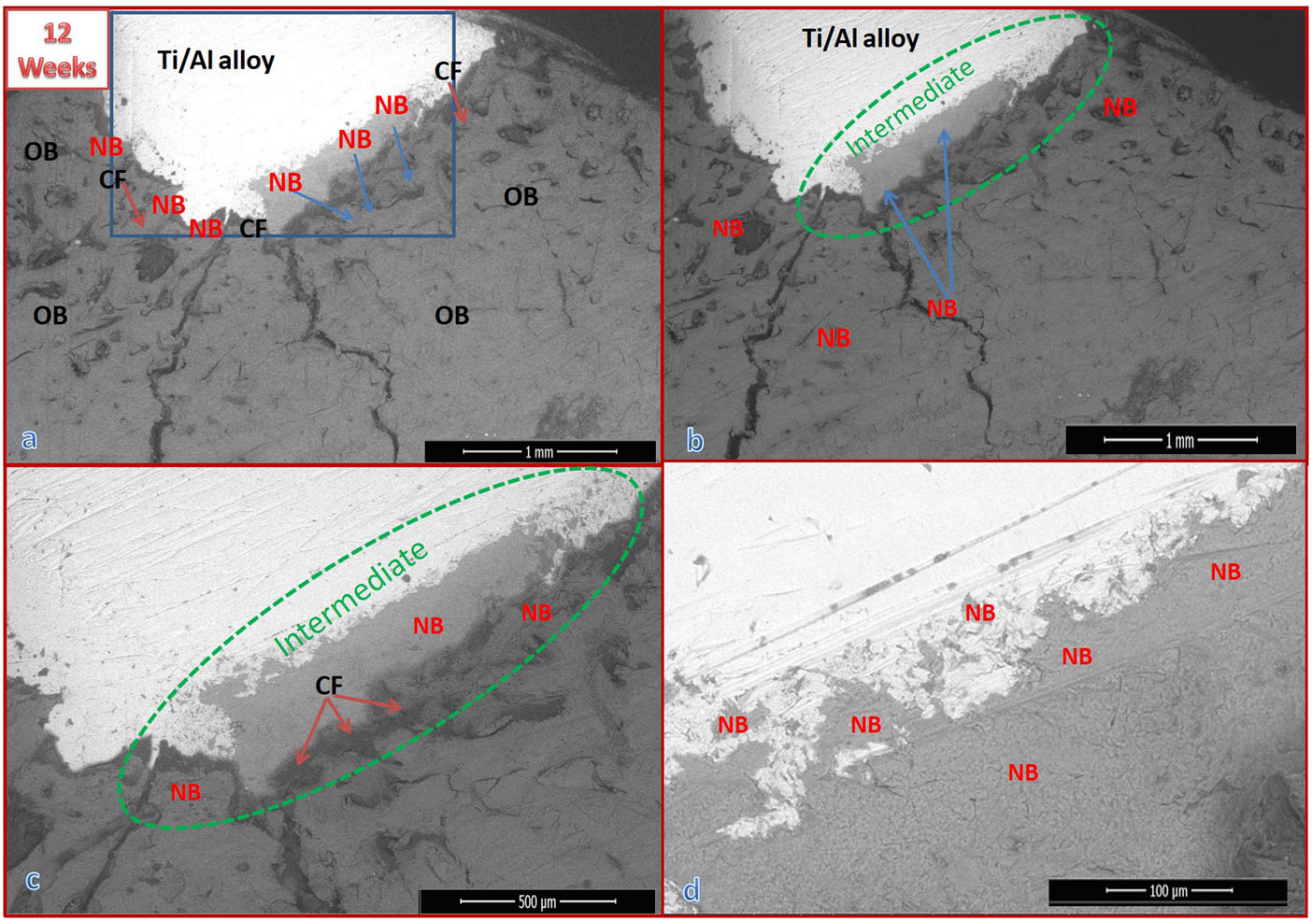
SEM micrographs with different magnifications for a cross-section of bone defect drilled in the dog femur and grafted with uncoated Ti/Al alloy for 12 weeks: **a** at ×60 magnification, b at ×500 magnification, **c** at ×1000 magnification, and **d** at ×1200 magnification

**Fig. 7:**
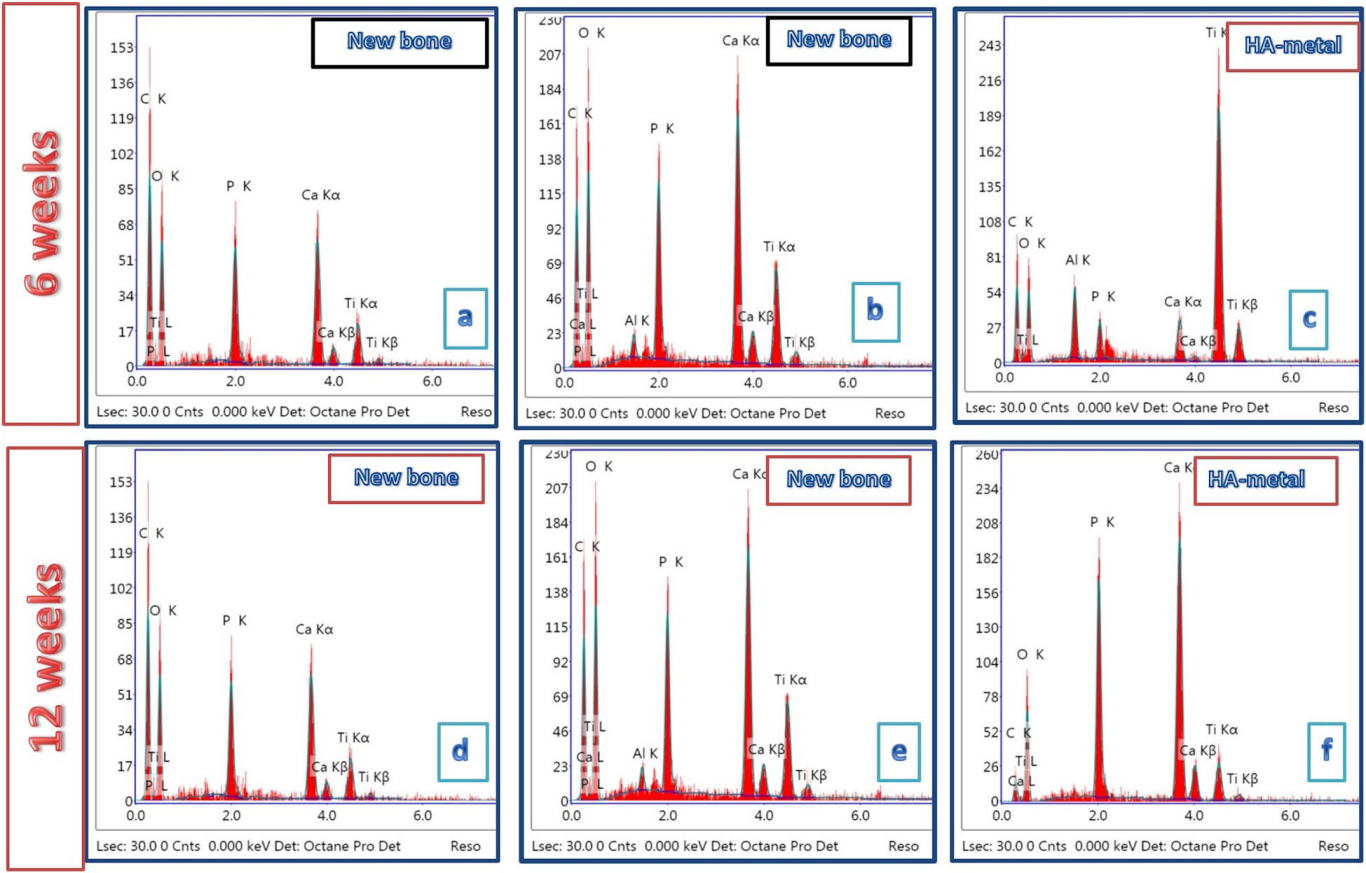
EDX analysis at 12 weeks post surgery: **a**–**c** (control group GI), Ti/Al alloy coated (bHA) GIII) for 6 weeks and (bHA) coat metal alloy, **d**–**f** (control group GII), TI/Al/ alloy coated with (bHA) GIV)

Figure 5a–d presents the SEM micrographs at different magnifications for a cross-section of the implanted bone defect created in the dog femurs. The bones were grafted with uncoated Ti/Al alloy (control group), and the dogs were sacrificed at 6 weeks post surgery. The defect area edges had appeared well, and the hole defect area (blue area) was still opened and was bridged to the original bone with uncalcified bone tissue (collagen fibers) (black area edges) red box, around and also on the alloy implant Fig. 6a. Analysis of the images in Fig. 6 revealed that the defect was not quite cured. Newly formed bone NB was discerned on the implant and also on the intermediate layer between the alloy and the old bone (OB). A noticeable number of fragments of the new bone were observed together with RBCs (marked by a yellow circle), indicating the bioactivity of the alloy Fig. 5c. The EDX pattern shown in Fig. 7a indicated that the newly formed bone (blue arrow) within the defect was not yet well mineralized. The Ca/P molar ratio was 1.81.

##### Group III results

**Fig. 8:**
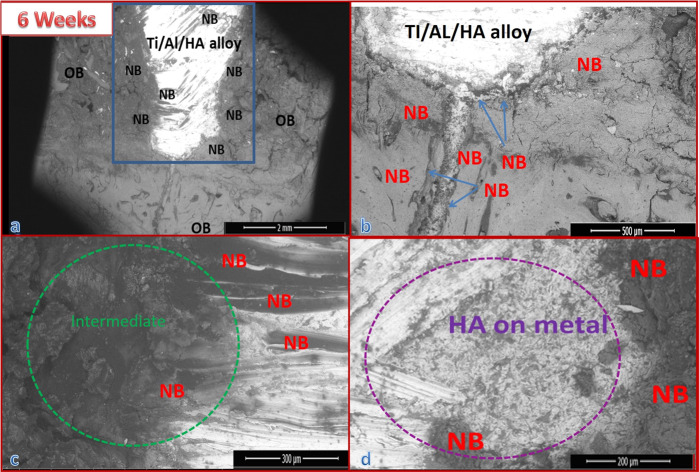
SEM micrographs with different magnifications for a cross-section of bone defect drilled in a dog’s femur and grafted with bHA-coated Ti/Al alloy for 6 weeks: **a** at ×60 magnification, **b** at ×500 magnification, **c** at ×1000 magnification, and **d** at ×1200 magnification

Figure 8a–d shows the SEM micrographs for a cross-section of the implanted bone defect created in the dog femurs. The bone defect was grafted with bHA-coated Ti/Al alloy (treated group). The images show that the bone defect was nearly healed and that it was inhabited with newly formed bone. The bone marrow was still congested with fragments of the bHA that coated the implanted alloy. Most of these fragments were surrounded by newly formed bone (blue arrow) Fig. 8a. The green circle in Fig. 8c indicates the intermediate layer between the coated alloy and the newly formed biogenic bone. The EDX pattern for the newly formed bone covering the grafted alloy is shown in Fig. 7b. Our analysis revealed that the newly formed bone was mineralized at approximately the same level as the original bone (Ca/P molar ratio 1.89), indicating that it was still not well mineralized; the Ca/P ratio for the normal bone was 2.30, and that for the coated metal alloy was 1.60 Fig. 7c.

#### For 12 weeks post surgery

##### Group II results

Figure 6a–d shows SEM micrographs with different magnifications for a cross-section of the bone defect in the dog femurs. The defect was grafted for 12 weeks with uncoated Ti/Al alloy (control group). The green circle indicates the intermediate layer, the blue area indicates the hole defect area, and the blue arrow indicates the formation of new bone. In Fig. 6, it can be seen that the grafted bone was not entirely healed. The EDX pattern shown in Fig. 8d indicates that the newly formed bone inside the defect was not well mineralized, with a Ca/P molar ratio of 1.83.

##### Group IV results

**Fig. 9:**
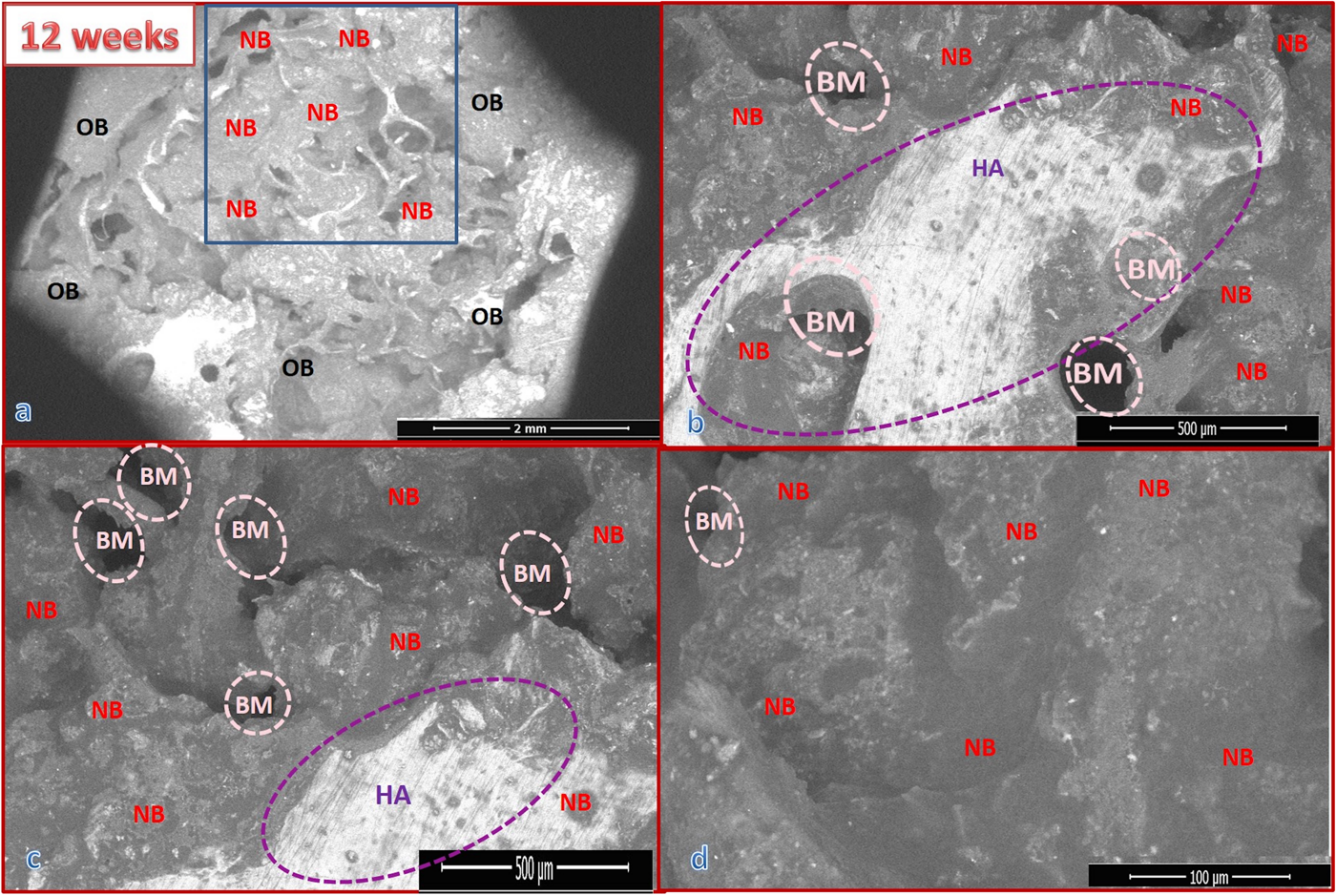
SEM micrographs with different magnifications for a cross-section of bone defect drilled in the dog femur and grafted with bHA-coated Ti/Al alloy for 12 weeks post surgery: **a** at ×60 magnification, **b** at ×200 magnification, **c** at ×300 magnification, and **d** at ×1200 magnification

Figure 9a–d displays the SEM micrographs for a cross-section of a dog femur bone defect that was implanted with a bHA-coated Ti/Al-alloy device for 12 weeks. The figure clearly indicates that the bone defect was completely recovered and that it was penetrated and filled by the newly formed bone. The hole defect area is identified by the blue shading. The bone marrow was still packed with some fragments of the HA coating of the implanted alloy, but most of these were surrounded by the newly formed bone [34]. The bHA-coated alloy showed its capability of inducing new bone not only in both the bone defect and throughout the bone marrow gap (the pink circle). The purple circle indicates residue of the bHA coating. The EDX patterns for the newly formed bone are shown in Fig. 7e. The pattern points to the newly formed bone being well mineralized compared with the original bone.

##### Histopathological findings

**Fig. 10:**
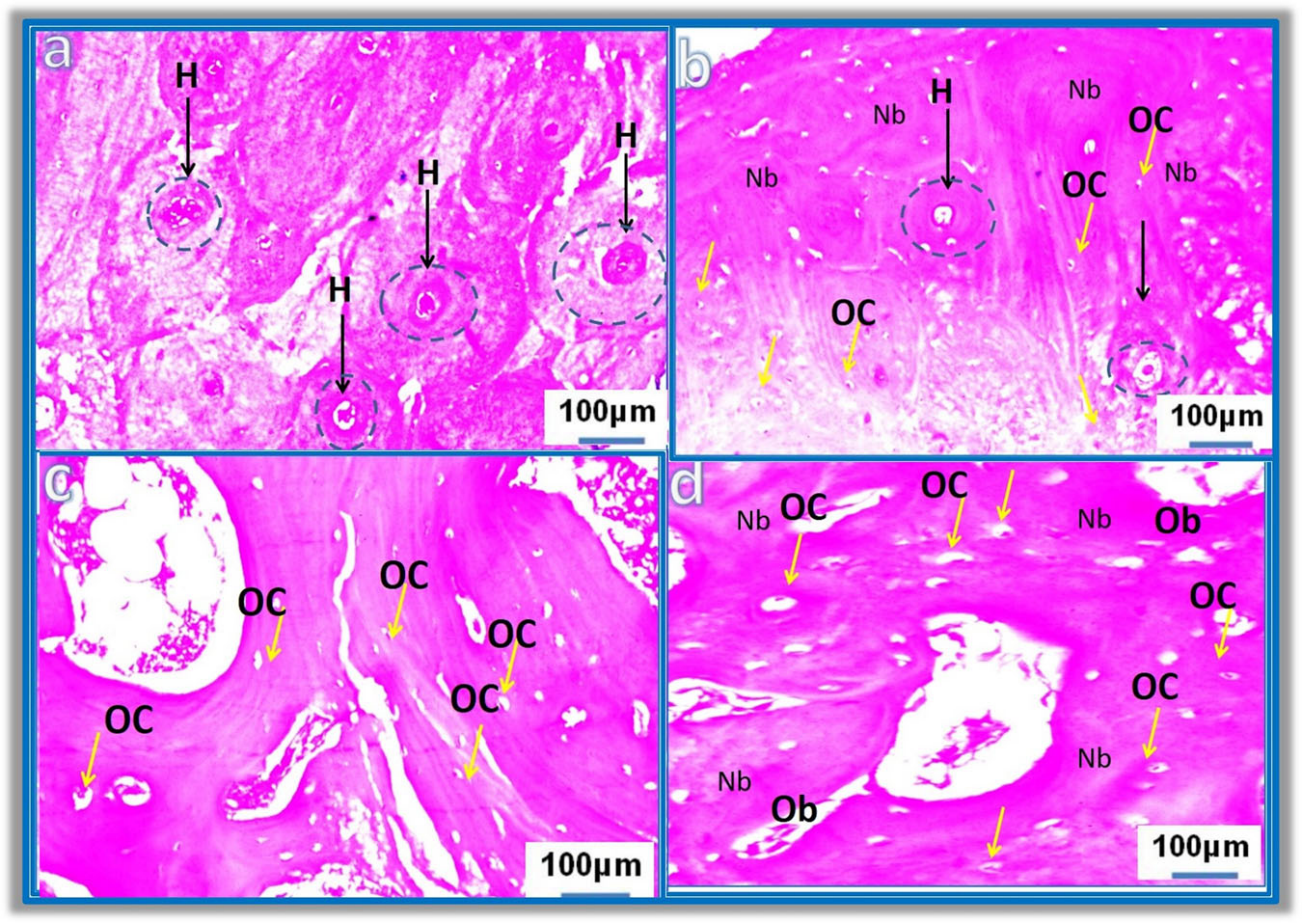
**a** Shows the histological picture of the femur bone for the dogs treated with uncoated Ti/Al alloy for 6 weeks (Group I, control), **b** Histological picture of the femur bone for the dogs treated with bHA-coated Ti/Al alloy for 6 weeks (Group III, treated), **c** Histological picture of the femur bone for the dogs treated with uncoated Ti/Al alloy for 12 weeks (Group II, control), **d** Histological picture of the femur bone for the dogs treated with bHA-coated Ti/Al alloy for 12 weeks (Group IV, treated)

Figure 10a shows some focal areas for Group 1 (control). The figure indicates a loss in the histological Haversian system (H, black arrows), (at magnification 100 µm). Figure 10b shows the histological picture of the femur bone for the dogs treated with bHA-coated Ti/Al alloy for 6 weeks (Group III, treated). The figure shows that most of the Haversian systems (H) had a normal histological structure (black arrows), with osteon canals (black arrows) containing blood vessels with well-stained osteocyte (OC, yellow arrows) (at magnification 100 µm). The histological picture for the femur bone of the dogs treated with uncoated Ti/Al alloy for 12 weeks (Group II, control) is shown in Fig. 10c. The figure shows multiple osteocytes (yellow arrows) and a normal histopathological structure (at magnification 100 µm). The histological picture of the femur bone for the dogs treated with bHA-coated Ti/Al alloy for 12 weeks (Group IV, treated) is shown in Fig. 10d. The figure shows large numbers of osteoblasts (Ob) surrounding the newly formed bone as well as a normal histopathological architecture (at magnification 100 µm).

## Discussion

The findings of the kidney function biochemical indicators are normal, which indicates that the released products of the coated alloy did not cause any renal failure. On the other hand, the coated implants led to a higher ALP activity (kidney function) than in the control group (Group II), which is attribute to the formation of new bone. Comparing the ALP values of the tested dogs with normal levels indicates that the tested dogs had normal kidney function as the normal ALP range for dogs is [20–200 U/L] [28].

The results of the hematology showed that the changes in the blood parameters were unremarkable, proving that neither the implantation of the uncoated nor the coated alloy had an immediate effect on these parameters. It is to be stated that the observed increments were natural and a result of the effects of the operation on the dogs’ health, the healing process and the formation of new bone in the defect area. Overall, the data presented in the results section indicate that neither the implantation of the uncoated nor the coated alloy had toxicity effects on the blood of the dogs in our sample.

**Fig. 11:**
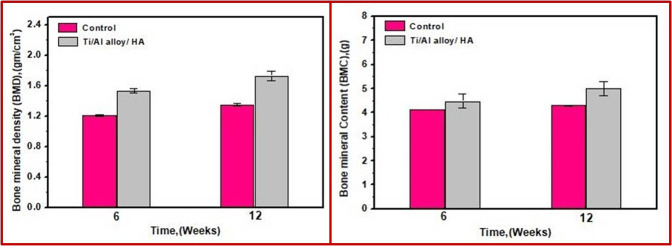
Mean values for bone mineral density (BMD) (g/cm^2^) and bone mineral content (BMC) (g) for the control groups and the treated groups

The observed bone density differences between the grafted bone and the control bones may be attributed to several factors. One of them is that several portions of implanted bHA were still found in the area of the hole, which appeared by yellow color in Fig. 3 (the appearance of yellow color indicates high BMD). Another factor is that the new bone tissue had grown and expanded considerably further through the defect area after 12 weeks post surgery compared with 6 weeks. Additionally, newly developed bone becomes more mineralized. The results indicated that the BMD for Group IV (bHA-coated alloy after 12 weeks) defects was very close to that of the healthy bone. Furthermore, there was no significant difference between the treated groups and the control groups, as shown in Figs. 3 and 11.

The results of the radioactive analysis articulated that the increased radiopacity of the bone opacity filling the entire medullary cavity surrounding the coated implants is a reflection of its increased osteogenesis produced by the bHA-coated implanted rods, and that the radiopacity around the implant, which filling the marrow cavity, is due to the bone formation.

After 6 weeks of implantation the analysis of the SEM images for the uncoated alloy indicates the presence of new bone fragments together with RBCs, which reveals that the defect was not quite cured, but at the same time proves the bioactivity of the alloy. The Ca/P molar ratio of the newly formed bone was 1.81 indicating that the newly formed bone within the defect was not yet well mineralized. On the other hand, the bone defect grafted with bHA-coated Ti/Al alloy was nearly healed and inhabited with newly formed bone. These findings show that the bHA-coated alloy was able to induce new bone generation not only in the induced bone defect but also throughout the bone marrow gap; this agrees with the BMD and radiography results.

After 12 weeks the bHA-coated alloy manifested its biocompatibility and capability of inducing new bone not only in the bone defect but also throughout the bone marrow gap. The Ca/P molar ratio of the new bone was 2.30, which was similar to the normal carbonated HA [35, 36]. We also note that, after 12 weeks, the newly formed bone induced by the bHA-coated alloy was more mineralized than that induced by Group II (control), the defect was completely cured, and the bone had the appearance of a normal bone.

The loss in the histological Haversian system of the control samples after 6 weeks of implantation can be attributed to the starting of the healing process in the hole defect area and the initial formation of new bone. Accordingly, it can be concluded that, after a 6-week implantation period, the dogs implanted with the uncoated alloy only showed a focal area of loss Haversion systems. However, the groups implanted with the bHA-coated alloy manifested multiple Haversion systems, reflecting a normal histopathological structure, complete healing of the defect area, and the formation of normal bone. This indicates that bHA accelerated the healing and remodeling of bone after the implantation of the alloy [37–39]. Furthermore, both Group III and Group IV displayed normal histopathological structures after 6 weeks of implantation (and 12 weeks for Group IV). These findings show agreement with the radiographic analysis and BMD results.

## Conclusions

- Based on the results presented above, it can be concluded that the degradation products of the bHA-coated Ti/Al alloy did not cause any liver disorder or kidney failure when implanted in the dogs. Both the liver and kidney functions of the dogs were normal, and the tumor-marker findings indicated that the implanted materials had no carcinogenic or inflammatory effects. The results also showed that the implants had no effect on the measured blood parameters, and the CBC findings were normal.
- Implantation of the bHA-coated alloy led to the release of calcium ions, which attached to natural collagen fibers and accelerated the nucleation of natural apatite (carbonated HA crystal) and the formation of mineralizing bone.
- From the radiographic analysis, the radiopacity of the bHA-coated Ti/Al alloy was higher than that of the uncoated alloy at 12 weeks post surgery, indicating that increased osteogenesis was produced by the bioceramic-coated implant.
- The results indicated that the BMD at the defect in Group IV (coated alloy) at 12 weeks was very close to that for the healthy bone.
- Finally, it can be concluded that the use of the bHA coating on the Ti/Al alloy enhanced and increased the natural processes that occur during the mineralization and regeneration of induced bone and that this coated alloy can be used safely as an advanced orthopedic implant device.
